# Prokaryotic and eukaryotic skin microbiota modifications triggered by *Leishmania* infection in localized Cutaneous Leishmaniasis

**DOI:** 10.1371/journal.pntd.0012029

**Published:** 2024-03-13

**Authors:** Jesús Jaimes, Luz Helena Patiño, Giovanny Herrera, Claudia Cruz, Julie Pérez, Camilo A. Correa-Cárdenas, Marina Muñoz, Juan David Ramírez

**Affiliations:** 1 Centro de Investigaciones en Microbiología y Biotecnología-UR (CIMBIUR), Facultad de Ciencias Naturales, Universidad del Rosario, Bogotá, Colombia; 2 Grupo de Investigación en Enfermedades Tropicales del Ejército (GINETEJ), Laboratorio de Referencia e Investigación, Dirección de Sanidad Ejército, Bogotá, Colombia; 3 Molecular Microbiology Laboratory, Department of Pathology, Molecular and Cell-based Medicine, Icahn School of Medicine at Mount Sinai, New York, New York, United States of America; Instituto de Ciências Biológicas, Universidade Federal de Minas Gerais, BRAZIL

## Abstract

Cutaneous Leishmaniasis (CL) is a tropical disease characterized by cutaneous ulcers, sometimes with satellite lesions and nodular lymphangitis. *Leishmania* parasites, transmitted by sandfly vectors, cause this widespread public health challenge affecting millions worldwide. CL’s complexity stems from diverse *Leishmania* species and intricate host interactions. Therefore, this study aims to shed light on the spatial-temporal distribution of *Leishmania* species and exploring the influence of skin microbiota on disease progression. We analyzed 40 samples from CL patients at three military bases across Colombia. Using Oxford Nanopore’s Heat Shock Protein 70 sequencing, we identified *Leishmania* species and profiled microbiota in CL lesions and corresponding healthy limbs. Illumina sequencing of *16S-rRNA* and *18S-rRNA* genes helped analyze prokaryotic and eukaryotic communities. Our research uncovered a spatial-temporal overlap between regions of high CL incidence and our sampling locations, indicating the coexistence of various *Leishmania* species. *L*. *naiffi* emerged as a noteworthy discovery. In addition, our study delved into the changes in skin microbiota associated with CL lesions sampled by scraping compared with healthy skin sampled by brushing of upper and lower limbs. We observed alterations in microbial diversity, both in prokaryotic and eukaryotic communities, within the lesioned areas, signifying the potential role of microbiota in CL pathogenesis. The significant increase in specific bacterial families, such as Staphylococcaceae and Streptococcaceae, within CL lesions indicates their contribution to local inflammation. In essence, our study contributes to the ongoing research into CL, highlighting the need for a multifaceted approach to decipher the intricate interactions between Leishmaniasis and the skin microbiota.

## Author summary

Cutaneous leishmaniasis is a parasitic disease transmitted by arthropods that affects mammals, including humans, leading to skin ulcers that can be disfiguring, weakness, and inflammatory states in the individual. This disease impacts on global public health, affecting vulnerable communities, particularly in tropical and subtropical regions endemic to this condition. Research related to cutaneous leishmaniasis not only enables the development of more effective prevention strategies or treatments but also contributes to the exploration of new therapeutic options. This investigation focuses on identifying the parasite species causing the disease in military patients in Colombia, describing how an individual may be infected by one or more species and how the populations of microorganisms (prokaryotes and eukaryotes) present on the skin are affected by the parasite’s presence. Some of these microorganisms have been linked to the exacerbation or improvement of other skin diseases, opening doors to new fields of study related to the illness.

## Introduction

Leishmaniasis encompasses a broad spectrum of clinical conditions caused by the obligate intracellular flagellated parasite, *Leishmania*. There are four recognized types of Leishmaniasis: cutaneous (CL), mucocutaneous (ML), visceral (VL) and post kala-azar dermal leishmaniasis (PKDL). It is estimated that approximately 12 million individuals worldwide are infected and 350 million at risk of acquiring the infection. The vast majority of cases of this infection are concentrated in six countries (Brazil, Colombia, Afghanistan, Iran, Algeria and Syria), where each clinical spectrum has been linked to the host’s immune response, to one or more of the 22 parasite species that can infect humans and/or the tissues involved in the infection [1].

Colombia boasts the second-highest number of documented *Leishmania* species (*L*. *panamensis*, *L*. *braziliensis*, *L*. *guyanensis*, *L*. *infantum chagasi*, *L*. *mexicana*, *L*. *lainsoni*, *L*. *amazonensis*, *L*. *colombiensis*, *L*. *equatoriensis*, *L*. *naiffi*, *L*. *lindenbergi*) [2–5]. Most reported cases are concentrated in rural areas, particularly among the military population residing in wild regions. The most common clinical manifestation is CL (95–98%), followed by ML in 1–4% and VL in 1–1.5%.

Regarding the predominant species in the country, *Leishmania (Viannia) panamensis* has been reported in 61.3% of the civilian population, while *Leishmania (Viannia) braziliensis* is prevalent in 95.4% of the military population [3,6,7]. In this context, individuals residing in endemic regions for the disease are at a high risk of contracting it [7]. Military personnel and other professions that involve work in wilderness areas face an even greater risk due to their activities and continued exposure in areas inhabited by vectors [7].

In recent years, microbiota studies have focused on exploring new approaches for managing chronic and infectious diseases. This is also the case for parasitic diseases such as leishmaniases. It is pivotal to consider the predominant microbiota at the phylum level in healthy skin, comprising Bacteroidetes, Actinobacteria, Proteobacteria and Firmicutes. Representative genera within these phyla include *Corynebacterium*, *Cutibacterium*, *Staphylococcus*, *Micrococcus*, *Actinomyces*, *Streptococcus* and *Prevotella* [8–14]. This information is crucial, particularly considering the observed effects of these bacterial communities in various inflammatory skin conditions, such as atopic dermatitis, psoriasis, suppurative hidradenitis and acne, among others. These conditions have demonstrated both beneficial and exacerbating effects linked to specific bacterial communities, contingent on the microorganism involved [15–21]. Notably, an increased abundance of *S*. *aureus* in atopic dermatitis and psoriasis correlates with heightened severity of clinical symptoms. Similarly, in conditions like acne, intra-species variations are evident, where the occurrence of inflammatory lesions is associated with an increased frequency of the AI phylotype of *Cutibacterium acnes* [1–5].

Several researchers emphasize the significance of skin microbiota in maintaining the overall health of the skin, the primary organ affected by the lesions caused by the *Leishmania* parasite. It is important to note that the presence of *Leishmania* and the subsequent ulceration led to a notable disruption in the skin’s microbiota composition. In these *Leishmania*-induced ulcers, the prevalent microbiota includes *Streptococcus*, *Staphylococcus*, *Fusobacterium* and other facultative anaerobes [13]. Expanding on this, studies conducted on murine models of various strains have revealed interesting insights. They suggest that in cases where the skin is sterile but with lesions characteristic of CL, there can be either a resistance to the disease’s progression or, conversely, the development of larger lesions [22, 23].

Regarding the inflammatory response, there is a discernible difference between organisms with sterile skin and those infected by *L*. *major*. In organisms with sterile skin, the initial response closely aligns with a Th1-type response. On the contrary, in infected organisms a Th2-type response prevails. This polarization is linked to larger lesions and more extensive tissue damage observed in histopathological analysis [22, 24]. Expanding upon this, studies conducted in both humans and mice have shed light on alterations in the predominant bacterial populations around and near the lesion in comparison to the healthy contralateral body zone. This shift is primarily characterized by an upsurge in the genera *Staphylococcus sp*. and *Streptococcus sp*. in response to *L*. *major* infection. [24].

Drawing from various studies conducted on human subjects, Salgado *et al*. reported a higher abundance of Firmicutes (54%), Actinobacteria (11.7%), Fusobacteria (11.6%), Proteobacteria (8.7%) and Bacteroidetes (5.1%) within lesions of patients from Brazil afflicted with CL caused by *L*. *braziliensis* across different body areas [25]. Additionally, Gimblet *et al*. observed alterations in bacterial diversity within lesions of Brazilian patients affected by CL due to *L*. *braziliensis*, where *Staphylococcus* and *Streptococcus* were predominant. They proposed that this dysbiosis amplifies inflammation, thereby potentially enhancing susceptibility to cutaneous diseases during *L*. *major* infection in murine models [24]. Recent studies, such as those by Ereqat *et al*. on patients from Saudi Arabia with lesions caused by *L*. *tropica* and *L*. *major* across various body regions, noted shifts in bacterial diversity marked by a prevalence of *Actinobacteria* and *Proteobacteria* [26]. Kaluarachchi and colleagues observed an increase in the Bacilli class, a rise in the relative abundance of Firmicutes genera and a decrease in Actinobacteria and Bacteroidetes in *L*. *donovani* lesions compared to the healthy contralateral skin [27]. In parallel, Amorim *et al*. highlighted the predominance of the *Staphylococcus spp*. genus in lesions caused by *L*. *braziliensis*, while also noting instances of *Corynebacterium*, *Streptococcus*, or *Arcanobacterium* predominance [28].

Nevertheless, investigations into the microbiota of CL lesions have primarily taken place in Brazil, focusing on *L*. *braziliensis* or old-world parasite species. Importantly, this knowledge gap persists for Colombia [24–28], one of the countries with the highest number of CL cases worldwide. The diversity of microorganisms presents in lesions caused by different *Leishmania* species, notably with *L*. *braziliensis* and *L*. *panamensis* in Colombia, remains poorly understood. Furthermore, previous studies have primarily centered on bacteria, overlooking potential involvement of other microorganisms, such as eukaryotes, in the inflammatory processes.

In this study, we comprehensively investigated the *Leishmania* species, as well as the bacterial and eukaryotic communities within lesions of military patients with CL. This research is especially significant given the high prevalence of this disease in Colombia, the limited research conducted on new world parasite species and the significantly impact of this disease in military forces due to their exposure to endemic areas [29,30]. To identify the *Leishmania* species, we employed next-generation sequencing, utilizing the Heat Shock Protein 70 (*HSP70*) marker [31–35]. Additionally, we discriminated the bacterial and eukaryotic communities using the V4 region of the *16S-rRNA* and *18S-rRNA* genes, respectively. Regarding the aforementioned, this study is exploratory and descriptive. We utilized convenience lesion sampling (sample by scraping). Based on the existing literature, we expect to observe an impact on the diversity of both prokaryotic and eukaryotic microorganisms compared to healthy skin (sampled by brushing) from the upper and lower limbs in this study. These variations may be influenced by additional factors such as the geographic sampling site, patient’s phototype, the specific body area sampled, the *Leishmania* species present in the lesion, ulcer size (categorized into quartiles), and parasitic load.

## Materials and methods

### Ethics statement

The present study was carried out after receiving approval from the Ethics Committee of the Colombian Armed Forces. In accordance with Resolution 8430 of 1993 from the Ministry of Health of Colombia, this study was classified as having a minimum level of risk. Adhering to national ethical guidelines and the Declaration of Helsinki, under Article No. 2043 from March 2017 [36], the samples were coded to ensure patient confidentiality. For each patient, epidemiological record forms for reporting cutaneous leishmaniasis were duly completed. Informed written consent for the use of samples in research was obtained as per the committee’s approved protocols.

### Study population and sample collection

In this study, we enrolled a total of 112 male participants aged between 18 and 32 years. Inclusion criteria dictated that these individuals had a prior diagnosis or demonstrated suspected cases of CL with associated lesions. They also met the probable case definition outlined in the Public Health Surveillance Protocol for Leishmaniasis [37]. Furthermore, they presented non-superinfected CL lesions on either their upper or lower limbs, with a lesion duration ranging from a minimum of one month to a maximum of 3 months. An additional criterion was that participants had not received any form of oral antibiotic treatment within the two months leading up to their study enrollment. The presence of systemic dermatological diseases or immunological conditions was considered as an exclusion criterion. Lastly, willing participation in the study was confirmed through informed written consent, signed voluntarily by the participants.

The samples were sourced from military bases located in Bonza, Medellín and Guaviare (S1 Fig), chosen due to their high case volumes. According to the Weekly Epidemiological Bulletin (Epidemiological Week 29 of 2023), a national cumulative total of 3,547 cases was anticipated, with 2,199 cases reported by that date. Specific case counts were gathered through direct communication for the years 2022 and 2023 (up to August) for these military bases: 451 and 357 cases for Bonza, 194 and 77 cases for Medellín and 191 and 19 cases for Guaviare, corresponding to the bases with most CL cases nationwide giving us support to select them for this sampling. Out of the 112 total samples collected, 54.46% (n = 61) were from the Bonza, 35.71% (n = 40) from Guaviare and 9.83% (n = 11) from Medellín. These samples were meticulously coded based on the collection site initials (B = Bonza, G = Guaviare, M = Medellín), the sample type (L = Skin Lesion, S = Healthy Skin) and a unique numerical identifier (e.g., BL-13) (S1 Fig).

For each participant enrolled in the study, a sample was obtained from the most recent or least progressed lesion. This involved carefully scraping the lesions present on the patient’s upper or lower limbs, following established protocols detailed in the "Manual of Procedures for Surveillance and Control of Leishmaniasis in the Americas" by the Pan American Health Organization [38]. To serve as a control of healthy skin, a sample by brushing was taken from an area on the contralateral limb with no lesions or dermatological alterations, using a Cepimax conical vaginal cytobrush. All procedures adhered to biosafety protocols and an aseptic solution was applied after sample collection.

The collected samples were placed in sterile 1.5 mL Eppendorf tubes, each containing 1 mL of RNA Later (Termo Fisher Scientific Inc.). Immediate storage at 6°C followed sample collection, maintaining this temperature until further processing in the laboratory for molecular detection of *Leishmania* [39]. Furthermore, comprehensive data were recorded, encompassing the geographical site of sample collection, the patients’ skin phototype, the specific body area from which the sample was taken and the size of the ulcers (S1 Table).

### DNA extraction

The samples stored in Eppendorf tubes underwent centrifugation at 13,000 rpm for 10 minutes to concentrate the sample. Following this step, the supernatant was carefully removed, and the resulting pellet was retained. DNA was extracted from these samples utilizing the DNeasy Blood & Tissue Kit (Qiagen, Germany) following the manufacturer’s protocol, ensuring an optimal DNA quality and concentration for subsequent sequencing (≥ 5 ng/μL) [40].

The obtained DNA was divided into three vials. Two vials containing 30 μL each were allocated for microbiota identification, while the third vial containing 15 μL was reserved for *Leishmania* species detection and identification. To assess the quality and concentration of each DNA sample, a NanoDrop/2000/2000c spectrophotometer (Thermo Fisher Scientific, Massachusetts, USA) was employed. The criteria for optimal DNA quality were a 260/280 ratio between 1.8–2.0 and a 260/230 ratio between 1.8–2.5. Furthermore, the integrity of the extracted DNA was evaluated through 1.5% agarose gel electrophoresis.

### *Leishmania* detection and species identification

To detect *Leishmania* and estimate parasite load, we followed the real-time PCR (qPCR) protocol targeting the *18S-rRNA* gene, as outlined by Ramírez *et al*. in 2023 [41]. Specifically, we employed the following primers: 18SrDNAF 5’-GTACTGGGGCGTCAGAGGT-3’; 18SrDNAR 5’-TGGGTGTCATCGTTTGCAG-3’; and 18SrDNA Tq5’-FAM AATTCTTAGACCGCACCAAG-NFQ-MGB-3’. For *Leishmania* species identification, samples that tested positive in the initial qPCR underwent PCR using primers described by Hernandez et al. in 2014 [33]. This targeted the *HSP70* gene and we utilized the Phusion High-Fidelity PCR Master Mix (New England Biolabs) for amplification. Subsequently, we assessed the quality and integrity of the amplicon through 2% agarose gel electrophoresis, selecting samples with higher intensity bands falling within the 300–400 bp range.

Next, we proceeded to sequence the amplicons utilizing high-fidelity enzymes. In the initial step of library preparation, we utilized the NEBNext Ultra™ II End Repair/dA-Tailing Module kit. Following this, we performed barcode ligation using the NEBNext Ultra™ II Ligation Module kit, ONT Barcode Kit (EXP-NBD196) and adapter ligation using the NEBNext Quick Ligation Module kit and the Oxford Nanopore Technologies Ligation Kit (SQK-LSK109). Subsequently, we sequenced the constructed library on the ONT MinION using R.9.4 flow cells and MinKnow V.3.1.4 software. For bioinformatic analysis, we worked with the raw Fast5 files, using base calling to obtain Fastq files. These files were then demultiplexed using Guppy V3.1.5.

Following this, a merging procedure was conducted for reads that met the specified quality filters. These high-quality reads were then compared with a reference database generated from available sequences of different *Leishmania* species found in the Nucleotide section of PubMed. This database was carefully curated using the UGENE v.33.0 software. The objective was to taxonomically assign these high-quality reads for the precise detection of corresponding *Leishmania* species. To achieve this, a local BLASTn analysis was employed, utilizing a minimum identity threshold of 95% and an e-value of 10. Reads that matched with a relative abundance exceeding 5% (in relation to the total reads) were selected for further analysis. This stringent criterion aimed to mitigate potential errors arising from the sequencing process [42]. Additionally, we fine-tuned the relative abundances of the *Leishmania* species’ reads, considering the copy number of the *HSP70* gene in each species. This adjustment provided a more accurate representation of the abundance of each *Leishmania* species (S2 Table). Subsequently, all quantitative results were effectively visualized using RStudio version 3.6.1 [42].

### Skin microbiota analysis

DNA was extracted from both lesioned (Lesion) and healthy (Healthy) skin samples with a concentration of ≥5 ng/μL. A conventional PCR was conducted targeting the hypervariable V4 region of the *16S-rRNA* gene using the primers 515f/806r (5’-3’) GTGCCAGCMGCCGCGGTAA/GGACTACHVGGGTWTCTAAT, which generate an amplicon of approximately 292 bp. Additionally, the hypervariable V4 region of the *18S-rRNA* gene was targeted using primers 528f/706r (5’-3’) GCGGTAATTCCAGCTCCAA/AATCCRAGAATTTCACCTCT, resulting in an amplicon of approximately 272 bp [43]. These regions, characterized by medium variability but widespread conservation, allow for taxonomic resolution of microorganisms present in the samples and are therefore commonly utilized in microbiota studies [43]. The Phusion High-Fidelity PCR Master Mix (New England Biolabs) was utilized for the PCR. Subsequently, amplicon quality and integrity were assessed through 2% agarose gel electrophoresis. The amplicons were then sequenced using the Illumina NovaSeq PE250 platform, with paired-end reads of 250 bp length and an expected raw read depth of 100,000 reads. [44,45].

Initially, Illumina sequencing yielded raw sequences in FASTQ format, comprising both read1 (Forward) and read2 (Reverse). These sequences underwent rigorous quality filtering using FastQC [46] and MultiQC version 1.6 [47] analyses, adhering to predetermined quality parameters (Phred Score equal to or greater than 20). Furthermore, an in-depth quality assessment was conducted for demultiplexed sequences corresponding to the *16S-rRNA* and *18S-rRNA* markers, using FastQC and MultiQC, with a stringent Phred Score threshold of 30. Following this, the sequences underwent barcode removal for *16S-rRNA* and *18S-rRNA* sequences, utilizing the QIIME2 tool [48].

Taxonomic assignment of these sequences was carried out using the DADA2 version 1.16 Pipeline [49]. This pipeline included sequence truncation based on quality (QC), merging and removal of PCR chimeras and read errors. These steps collectively facilitated the construction of the Amplicon Sequence Variant (ASV) table. Taxonomic assignment was achieved by comparing the sequences with the SILVA version 138 database for *16S-rRNA* (prokaryotes) and the PR2 version 4.14.0 database for *18S-rRNA* (eukaryotes), This approach is particularly useful for identifying fungi and protists. The taxonomic assignment of ASVs was determined utilizing functions provided by the DADA2 package, considering a minimum Bootstrap of 50 [49].

### Statistical analysis

The composition of the most abundant Amplicon Sequence Variants (ASVs) was analyzed using the PhyloSeq (V1.26.1) program. We applied the Wilcoxon test for comparison, evaluated alpha diversity using Shannon and Simpson indices and assessed beta diversity through Principal Coordinates Analysis (PCoA). Kruskal-Wallis tests followed by Dunn’s test for multiple comparisons were employed, using RStudio packages, to compare various variables per sample. These variables included sample type, geographic sampling site, patient’s phototype, body area from which the sample was taken, *Leishmania sp*. species present in the lesion, ulcer size (categorized into quartiles: Q1 = 150mm, Q2 = 151-245mm, Q3 = 246-501mm, Q4 = 502-1963mm) and parasitic load (categorized into quartiles: Low (L) = 2,545 parasites/mL, Medium (M) = 2,546–14,398 parasites/mL, High (H) = 14,399–219,710 parasites/mL, Very High (VH) = 219,711–2,548,227 parasites/mL) (S2–S6 Figs). Subsequently, graphical representations of respective phyla and genera results were generated.

Furthermore, using the DESeq2 package and performing Analysis of Composition of Microbiomes (ANCOM), we determined the presence of differentially abundant groups [42]. Finally, we explored the correlation between the abundance values of prokaryotic genera and eukaryotic species with parasitic load using the Spearman correlation test.

## Results

### Sample collection locations and identified *Leishmania* species

Out of the 112 collected samples, 54.46% (n = 61) were obtained from the Bonza military base, 35.71% (n = 40) from Guaviare and 9.83% (n = 11) from Medellín. These samples were subsequently coded (S1 Fig). The 86.6% (n = 97) were identified as positive for *Leishmania* DNA. However, only samples that tested positive for both markers (*16S-rRNA* and *18S-rRNA* genes) and met Illumina sequencing criteria were selected for microbiota estimations, totaling 40 samples. Within these 40 samples, we conducted *HSP70* gene sequencing to identify the *Leishmania* species. On average, 2,723,558 reads were generated and sequences with an abundance greater than 5% of the total analyzed sequences were used. Notably, the chosen marker could not differentiate between *L*. *panamensis* and *L*. *guyanensis*, hence they are referred to as a *L*. *panamensis/guyanensis* complex.

**Fig 1:**
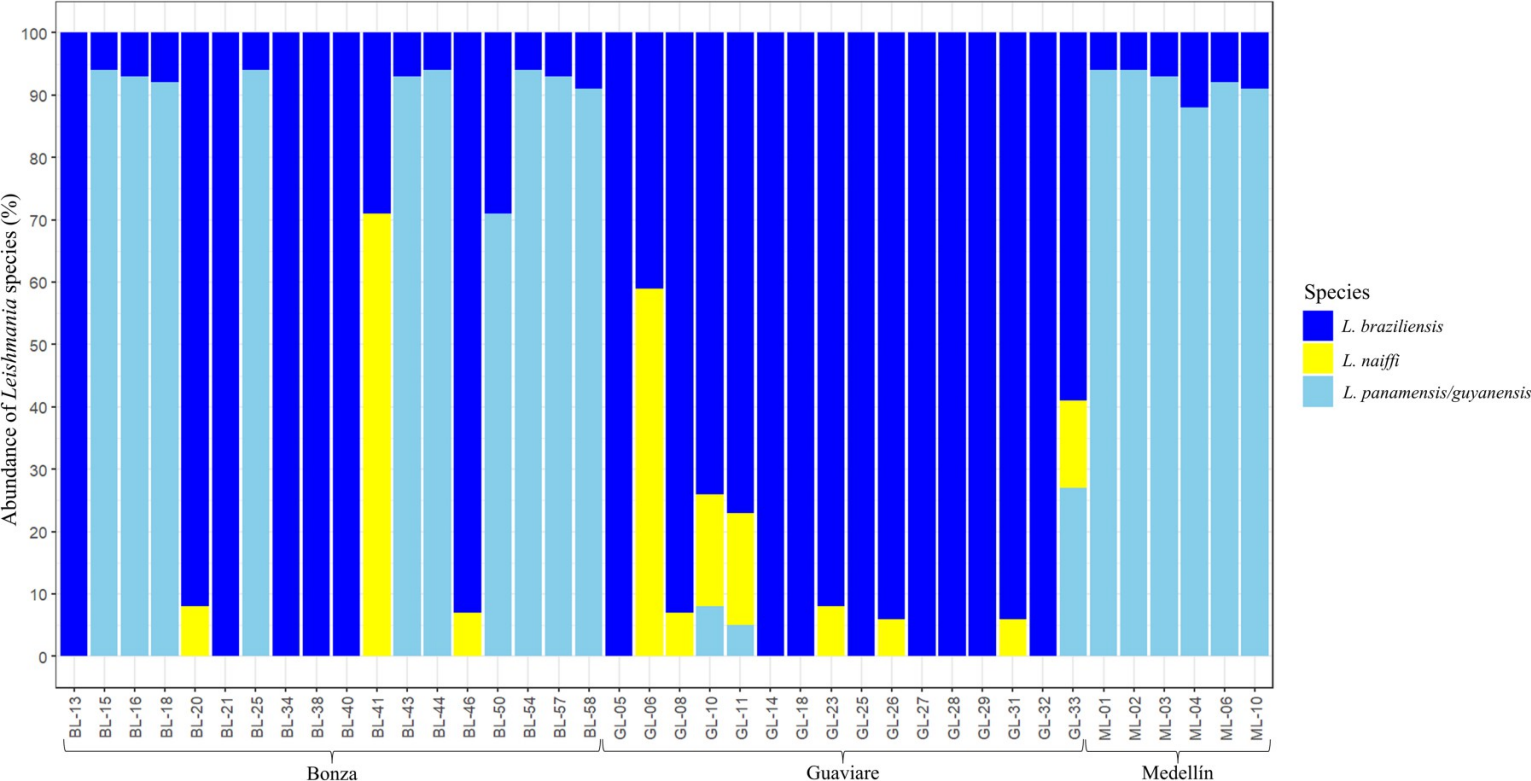
Relative abundance of *Leishmania* species. The abundance is expressed in percentage (%) related with the analyzed samples found in CL patients from three geographical locations in Colombia (Bonza, Guaviare, Medellín), focusing on those with an abundance greater than 5%.

Subsequently, it was found that 32.5% of the samples (n = 13) exhibited monoinfection with *L*. *braziliensis*. Meanwhile 40% of the samples showed coinfection involving *L*. *braziliensis* and *L*. *panamensis/guyanensis* complex. Additionally, 20% of the samples displayed coinfection between *L*. *braziliensis* and *L*. *naiffi* and 7.5% (n = 3) had coinfection involving *L*. *braziliensis–L*. *naiffi—L*. *panamensis/guyanensis* complex (Fig 1).

### Alpha and beta diversity in *Leishmania* infection

**Fig 2:**
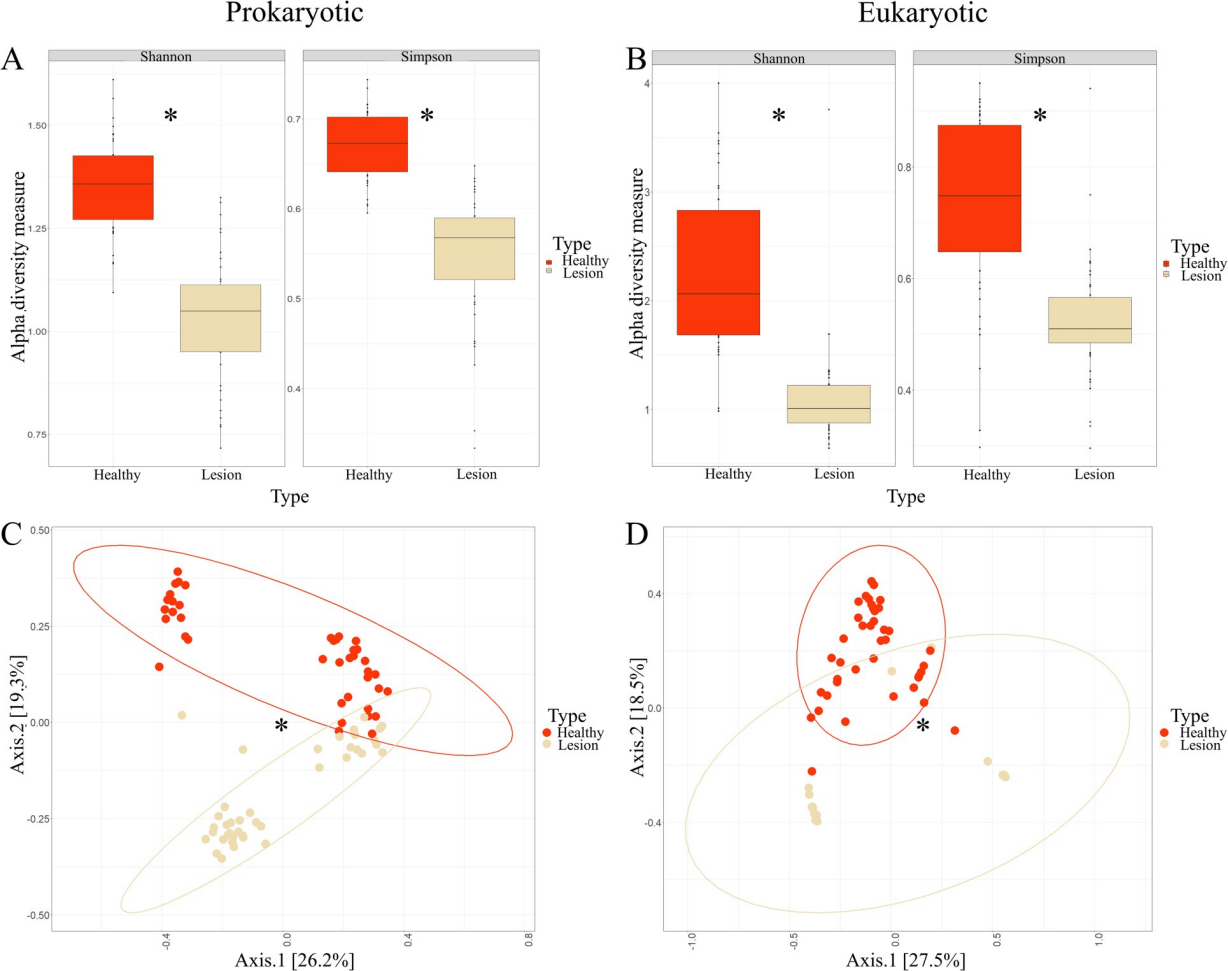
Alpha and beta diversity indices of prokaryotes and eukaryotes by sample type. Alpha diversity indices of prokaryotes (A) and eukaryotes (B) based on the sample type (healthy skin (Healthy) or the lesion (Lesion)). This is followed by beta diversity of prokaryotes (C) and eukaryotes (D) based on the sample type. (*) denotes significant differences between groups.

The results revealed a statistically significant reduction in alpha diversity for both the Shannon index (p = 2.924e-10) and the Simpson index (p = 1.619e-08) in the prokaryotic populations of the lesion samples (Fig 2A). Similarly, there was a decrease in both indices (Shannon p = 5.719e-15; Simpson p = 1.484e-08) for eukaryotes in the lesion samples compared to those from the healthy contralateral skin (Fig 2B). However, no significant differences were found when assessing alpha diversity indices of prokaryotes and eukaryotes based on the geographic site of sample collection, patient phototype, body area where the sample was taken, *Leishmania* species present in the lesion, ulcer size divided into quartiles (Q1 = 150mm, Q2 = 151-245mm, Q3 = 246-501mm, Q4 = 502-1963mm), or parasite load (Low (L) = 2,545 parasites/mL, Medium (M) = 2,546–14,398 parasites/mL, High (H) = 14,399–219,710 parasites/mL, Very High (VH) = 219,711–2’548,227 parasites/mL) (S2–S6 Figs).

In terms of beta diversity, distinctive patterns are observed, enabling spatial grouping among individuals from different categories for both prokaryotes (p = 0.001) and eukaryotes (p = 0.001) (Fig 2C and 2D). Conversely, no significant differences were noted when assessing beta diversity indices concerning the geographical location of sample collection, patient phototype, sampled body region, *Leishmania* species in the lesion, ulcer size categorized into quartiles, or parasite load (S7–S11 Figs).

### Taxonomic composition of the microbiota

**Fig 3:**
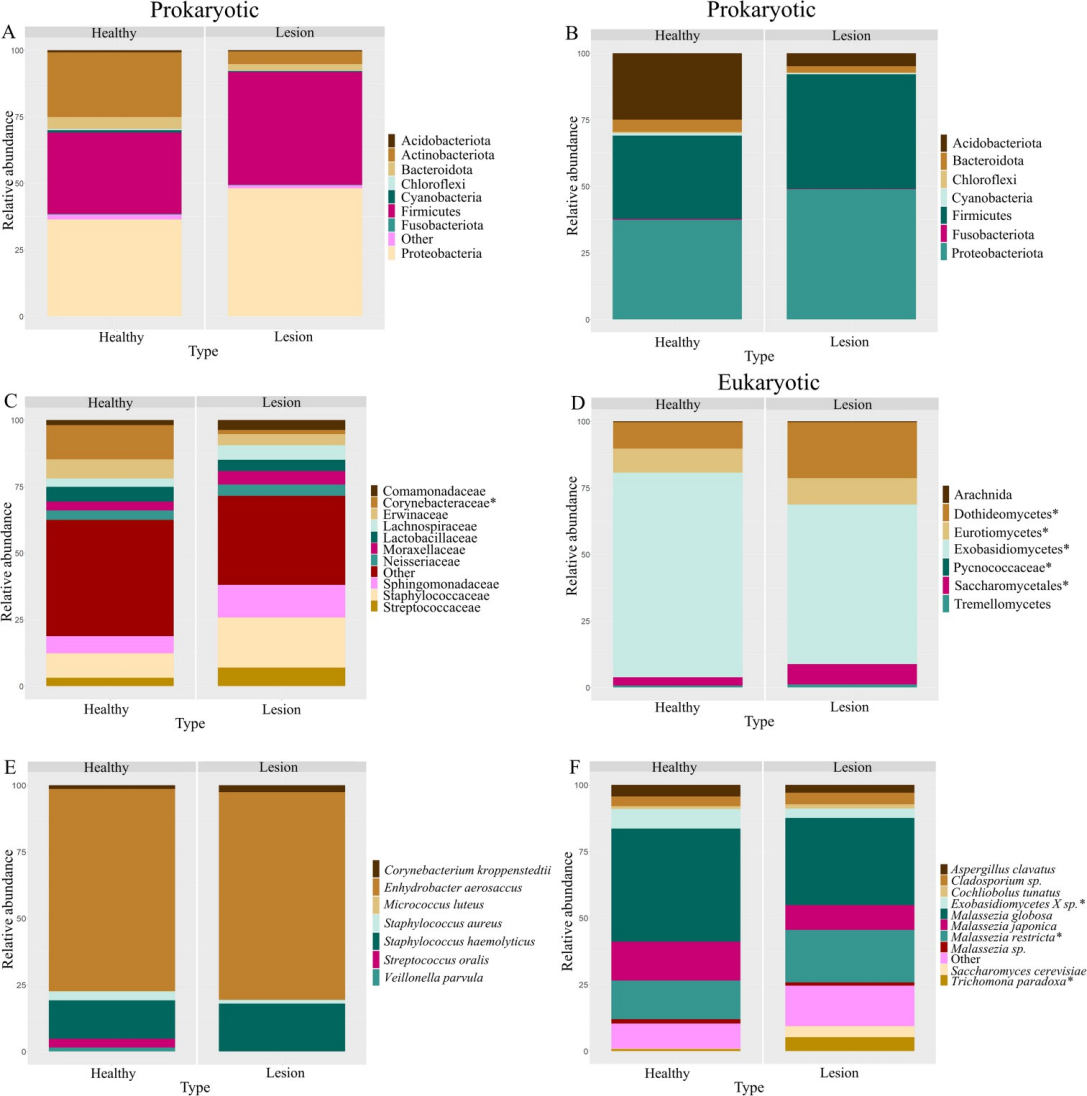
Relative abundance of prokaryotic and eukaryotic phyla, families, and species. Boxplots illustrating changes in the relative abundance of prokaryotic phyla within samples categorized by healthy skin (Healthy) and lesion skin (Lesion), both without (A) and with the subgroup (B) of the most abundant species found on the skin according to the literature. This is followed by an analysis of changes in the composition of prokaryotic (C) and eukaryotic (D) families. Finally, alterations in relative abundance are demonstrated for species using the subgroup approach for both prokaryotes (E) and eukaryotes (F). (*) denote statistically significant differences between groups.

**Fig 4:**
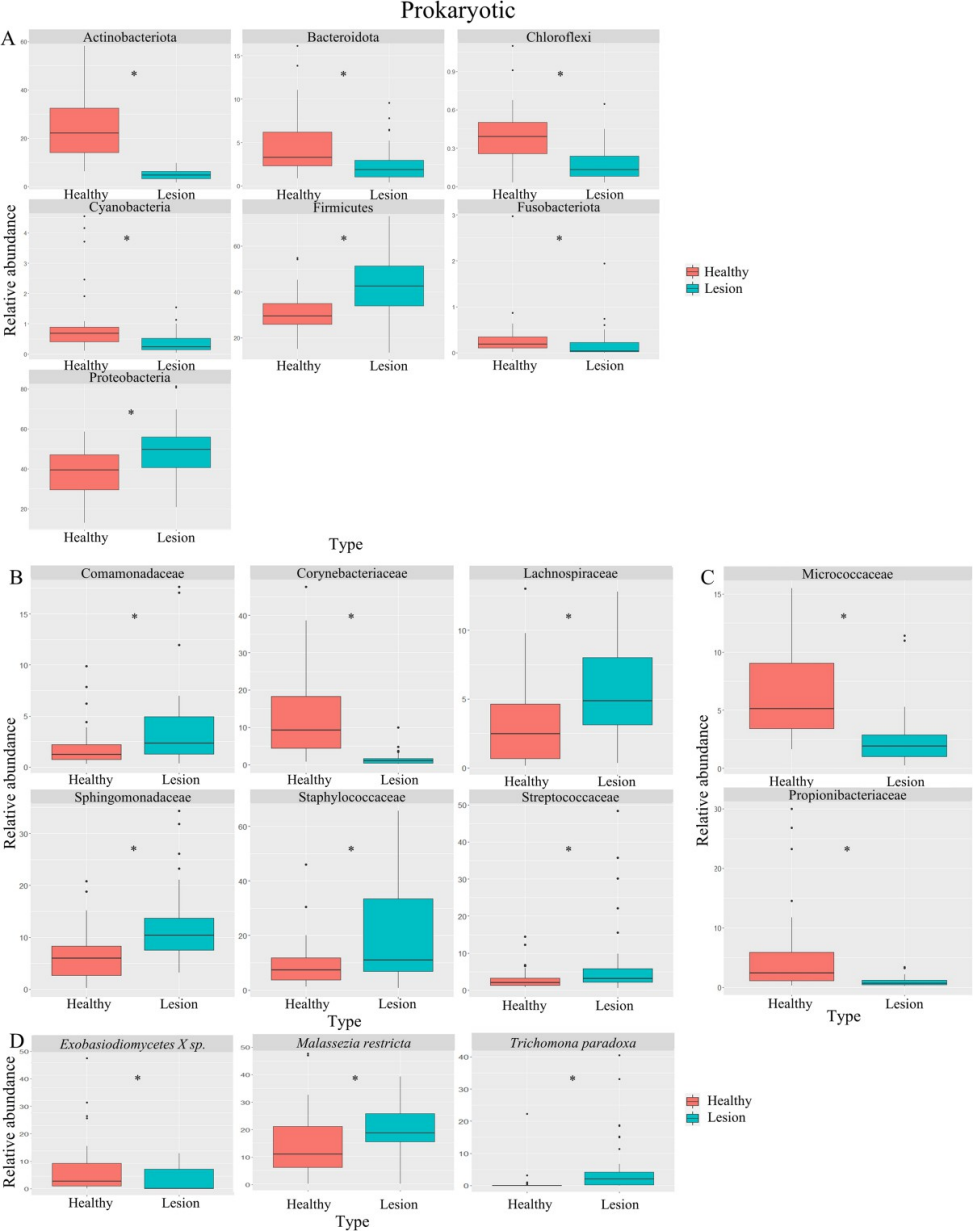
Relative abundance of sub-grouped prokaryotic and eukaryotic families and species. (A). Boxplots presenting alterations in the relative abundance of prokaryotic phyla within samples grouped into healthy skin (Healthy) and lesion skin (Lesion). Subsequently, the relative abundance of prokaryotic families is displayed, both without (B) and with the subgroup (C) of the most abundant species found on the skin according to the literature. Lastly, shifts in microbiota composition are portrayed for species from the selected subgroup of eukaryotes (D). (*) signify statistically significant differences between groups.

134,720 (min. 67,184, max. 149,458) reads for the *16S-rRNA* marker and 125,650 (min. 14,501, max. 179,867) reads for the *18S-rRNA* marker were sequenced. Samples were categorized into two groups: healthy skin (Healthy) and skin with a lesion or ulcer from LCL (Lesion). We first analyzed the microbiota composition at the phylum level for prokaryotes (Figs 3A and 3B and 4A) [10,50–52]. We observed a significant increase in Firmicutes (p = 3.795e-05) and Proteobacteria (p = 6.492e-04) phyla in lesioned skin, while Actinobacteria (p = 2.2e-16), Bacteroidetes (p = 1.367e-04), Chloroflexi (p = 1.398e-05), Cyanobacteria (p = 4.353e-06) and Fusobacteria (p = 4.525e-04) phyla showed a significant decrease (Figs 3A and 3B and 4A).

Furthermore, we delved into the microbiota composition at the family level for both prokaryotes (Figs 3C, 4B and 4C) and eukaryotes (Fig 3D) [10,50–52]. In prokaryotes, lesioned skin exhibited an increase in Comamonadaceae (p = 2.112e-03), Lachnospiraceae (p = 2.758e-05), Sphingomonadaceae (p = 1.468e-05), Staphylococcaceae (p = 5.4e-03) and Streptococcaceae (p = 1.266e-02) families and a decrease in Corynebacteriaceae family (p = 5.303e-14). Focusing on families commonly found in healthy skin, we observed a decrease in Micrococcaceae (p = 3.811e-09) and Propionibacteriaceae (p = 5.341e-08) abundance (Fig 4C). As for eukaryotes, the lesioned skin displayed a significant increase in Dothideomycetes (p = 1.204e-04), Eurotiomycetes (p = 2.599e-02), Saccharomycetales (p = 2.492e-05) and Picnococcaceae (p = 3.595e-04), with a decrease in Exobasidiomycetes (p = 6.74e-04) (Fig 4D).

Lastly, we examined the microbiota composition at the species level, focusing on commonly reported species in healthy skin [10,50–52]. In prokaryotes, there were no significant differences in lesioned skin compared to contralateral healthy skin (S12 Fig). Conversely, in eukaryotes, we observed an increase in *Malassezia restricta* (p = 5.421e-03) and *Trichomonas paradoxa* (p = 1.821e-05) abundance and a decrease in *Exobasidiomycetes sp*. (p = 1.22e-02) abundance (Fig 4D).

### Differential abundance analysis

**Fig 5:**
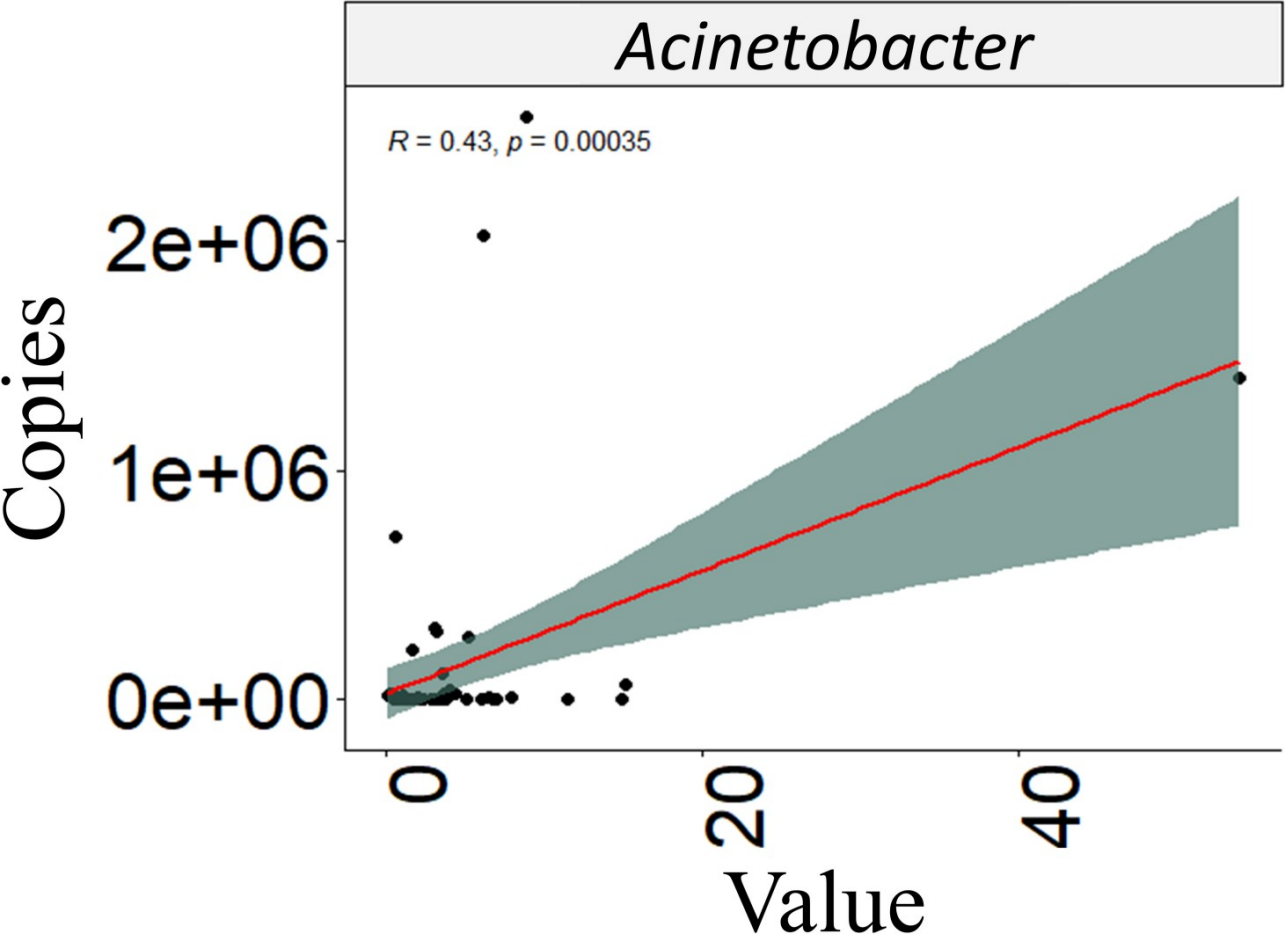
Correlation analysis between prokaryotic genus and parasitic load. Correlation analysis showing the abundance values of the genus *Acinetobacter*, the only significant genus in this analysis. Spearman’s correlation test was applied, and the resulting coefficient (R) and p-value are clearly indicated.

We conducted an analysis of differential abundances utilizing the Deseq2 package and an analysis of microbiome composition (ANCOM), (S13–S14 Figs). When employing lesioned skin as the reference group, the Deseq2 analysis uncovered an increase in abundance for the *Firmicutes* and *Proteobacteria* phyla, owing to positive rate changes (S13 Fig). Conversely, other phyla exhibited a decrease in abundance due to negative rate changes (S13 Fig). Within the ANCOM results, a set of species exhibited significant differential abundances, with prominent species including *Clostridium sp*., *Eubacterium sp*. and *Aerococcus sp*. (S14 Fig and S3 Table). Lastly, we explored the correlation between abundance values of prokaryotic genera, eukaryotic species and parasitic load using the Spearman correlation test. Surprisingly, we found only a weak statistically significant correlation for *Acinetobacter*. However, it is worth noting that no significant correlation was observed between these variables overall (Fig 5).

## Discussion

In this study, we observed a spatial-temporal overlap of the sampling areas with three regions of high disease incidence (S1 Fig). Our findings align with prior research who emphasized the preeminence of *L*. *braziliensis* as the species with the highest relative abundance among Colombian military personnel in rural regions (Fig 1). Additionally, we noted a heightened relative abundance of *L*. *naiffi* compared to the aforementioned studies [2,53]. However, it is essential to underscore the limitation posed by the relatively small sample size and the oversampling in Bonza (Center of the country) in this study, potentially leading to an overrepresentation of *L*. *naiffi*. Further studies employing a statistically significant sample size and distribution are warranted to comprehend the spatial and temporal distribution of *Leishmania* species within the military population (Fig 1).

Currently, co-infection events in both cutaneous and visceral leishmaniasis in Colombia have been previously reported [53,54]. These events were identified based on species’ relative abundances exceeding 3%. However, in our study, we applied a more stringent threshold of 5% to mitigate potential sequencing errors. Regarding co-infection events, Patiño et al. noted that 80% of cases exhibited *L*. *braziliensis*, 42% had *L*. *panamensis*, 6% presented *L*. *amazonensis*, *L*. *naiffi* and *L*. *infantum* each, while 4% demonstrated *L*. *mexicana* [53]. In contrast, our findings revealed that 100% of samples with co-infection events showed the presence of *L*. *braziliensis*. The *L*. *panamensis/guyanensis* complex was observed in 47.5% of cases and *L*. *naiffi* in 27.5% (Fig 1). Notably, *L*. *naiffi*, a species reported in the last 15 years in northern South American countries, appears to be increasingly prevalent in rural settings [55]. While clinical manifestations associated with this species are generally mild and self-resolving due to their low severity [56,57], reported cases are scarce. We hypothesize that co-infection events occur during instances of competition among parasite species, leading them to colonize distinct zones within the vector’s gut. This phenomenon has been demonstrated by Alexandre et al. (2022) in experimental coinfections involving *Lutzomya migoinei* and *Lutzomya longipalpis*. In this study, *L*. *infantum* was found throughout all midgut regions (suprapylarian development), while *L*. *braziliensis* concentrated in the hindgut and the abdominal midgut (peripylarian development) [58]. The significance of this lies in the potential occurrence of such co-infection events in nature, particularly when vectors feed on reservoirs infected with different parasite species. This scenario becomes critical as it might coincide with instances of reservoirs being coinfected with two or more parasite species during the same feeding moment. Nonetheless, the impact of species co-infection on the clinical outcomes of LCL remains poorly understood. Future studies should consider investigating and testing this premise to gain a more comprehensive understanding of its implications.

Regarding alterations in skin microbiota in LCL lesions compared to healthy skin, various studies have consistently observed a significant decrease in both alpha and beta diversity, encompassing prokaryotes and eukaryotes, a pattern in line with the findings of this study, evident in both domains (Fig 2A–2D). When examining at the phylum level, our results align with prior research, demonstrating an increase in the relative abundance of Firmicutes and Proteobacteria (Figs 3A and 3B and 4A and Table 1). Conversely, Salgado *et al*. reported an increase in Actinobacteriota and Bacteroidota abundance in lesions caused by *L*. *braziliensis* and Ereqat *et al*. observed similar trends in lesions caused by *L*. *tropica* and *L*. *major* [25,26]. However, Kaluarachchi *et al*., in agreement with our findings, described a decrease in these two phyla [25–27] (Figs 3A and 3B and 4A and Table 1). It is crucial to highlight that comparable reductions in these phyla have been noted in microbiota analysis of other ulcerative skin conditions, such as diabetic foot ulcers, suggesting localized structural and inflammatory alterations in affected areas [59,60]. As for other phyla experiencing a significant decline in abundance, including Actinobacteriota, Chloroflexi and Cyanobacteria, these microorganisms are recognized for their potential impactful roles [61–63]. Furthermore, the Fusobacteriota phylum is commonly associated with gastrointestinal pathologies, particularly of a tumoral nature [64,65]. Salgado *et al*. also reported an increase in the abundance of Fusobacteriota in response to *L*. *braziliensis* infection [25].

**Table 1:** Cutaneous Leishmaniasis and microbiota correlation consensus. Showing different studies and their different findings relating CL disease with prokaryote microbiota associations compared with our study results.

Taxonomic Classification		Microorganisms	Changes in Abundance in this study	Changes in Abundance in other studies	Association	*Leishmania* species	Reference
Prokaryotes	Phylum	Firmicutes	Increase	Increase	Not mentioned	*L*. *braziliensis*	[25]
			Increase	Increase	Not mentioned	*L*. *donovani*	[27]
		Proteobacteria	Increase	Increase	Not mentioned	*L*. *braziliensis*	[25]
			Increase	Increase	Not mentioned	*L*. *tropica; L*. *major*	[26]
		Actinobacteriota	Decrease	Increase	Not mentioned	*L*. *braziliensis*	[25]
			Decrease	Increase	Not mentioned	*L*. *tropica; L*. *major*	[26]
			Decrease	Decrease	Not mentioned	*L*. *donovani*	[27]
		Bacteroidota	Decrease	Increase	Not mentioned	*L*. *braziliensis*	[25]
			Decrease	Decrease	Not mentioned	*L*. *donovani*	[27]
	Class	Bacilli	Not analyzed	Increase	Wet, high parasitic load & large lesions (>2cm)	*L*. *donovani*	[27]
	Family	Staphylococcaceae	Increase	Increase	Increased local inflammation	*L*. *major*	[24]
			Increase	Increase	Increased local inflammation	*L*. *braziliensis*	[24]
			Increase	Increase	Not mentioned	*L*. *braziliensis*	[25]
			Increase	Increase	Increased local inflammation, delayed lesion healing when treated	*L*. *braziliensis*	[28]
		Streptococcaceae	Increase	Increase	Increased local inflammation	*L*. *major*	[24]
			Increase	Increase	Increased local inflammation	*L*. *braziliensis*	[24]
			Increase	Increase	Not mentioned	*L*. *braziliensis*	[28]
		Corynebacteraceae	Decrease	Increase	Large lesions (>2cm)	*L*. *donovani*	[27]
			Decrease	Increase	Not mentioned	*L*. *braziliensis*	[28]
	Genus	*Trabusiella*	Not significative	Increase	High parasite load lesions	*L*. *donovani*	[27]
		*Micrococcus*		Increase	Wet, high parasitic load & large lesions (>2cm)	*L*. *donovani*	[27]
				Decrease	Not mentioned	*L*. *braziliensis*	[28]
		*Corynebacterium*		Increase	Wet, high parasitic load & large lesions (>2cm)	*L*. *donovani*	[27]
		*Arcanobacterium spp*.		Increase	Inflammatory gene expression	*L*. *braziliensis*	[28]
				Increase	Not mentioned	*L*. *braziliensis*	[25]
				Increase	Small lesions (<2cm)	*L*. *donovani*	[27]
	Species	*Staphylococcus aureus*	Not analyzed	Increase	Increased local inflammation and lesion severity in mice	*L*. *braziliensis*	[24]
				Increase	Not mentioned	*L*. *tropica; L*. *major*	[26]
		*Streptococcus sp*.		Increase	Not mentioned	*L*. *braziliensis*	[25]
					Increased local inflammation and lesion severity in mice	*L*. *braziliensis*	[24]
		*Proteus mirabilis*		Increase	Not mentioned	*L*. *tropica; L*. *major*	[26]
		*Cutibacterium acnes*		Increase	Not mentioned	*L*. *tropica; L*. *major*	[26]

When considering the families of microorganisms with significant differences in abundance, it is crucial to highlight extensive research showing an increase in Staphylococcaceae and Streptococcaceae families within skin lesions. In this study, these families also appeared in higher abundance on the lesioned skin (Figs 3B and 4B) and have been primarily associated with a noticeable increase in local inflammation, suggesting their contribution to the characteristic inflammatory processes of LCL lesions [24,28] (Table 1). It is important to note that this abundance increase might be mediated by specific virulence factors present in these communities, as they express genes that protect them against Antimicrobial Peptides (AMPs) [66–69]. Additionally, it is relevant to consider that members of the Staphylococcaceae family can induce the activation of a Th1/Th17 immune response simultaneously with local dendritic cells, directly relating to the observed changes in the local inflammatory response [66,67,70]. This process appears to be mediated by the recruitment of neutrophils and interleukin-1β (IL-1β)-dependent [55–57,71]. On the other hand, it is essential to mention the role of *S*. *aureus* in this context [66]. Although no significant differences were identified concerning this genus in this study (Fig 3E), it has often been associated with the deterioration or exacerbation of immunologically derived skin diseases [16,18,19]. Conversely, *S*. *epidermidis* has also been shown to promote the generation of Th17 cells [72,73]. This leads us to consider that the presence of facultative anaerobes could be an adverse prognostic factor in the skin wound recovery process [74,75].

In regard to the Staphylococcaceae and Streptococcaceae families, which hold significant clinical relevance, there have been limited reports regarding the antibiotic resistance and sensitivity profiles of bacteria in CL lesions. Isaac-Márquez and Lezama-Dávila extensively evaluated antibiotic resistance patterns for common Gram-positive and Gram-negative bacteria in Chiclero ulcer skin lesions (*L*. *mexicana* infection) [76]. Their findings revealed resistance in *Staphylococcus* species, including *S*. *aureus*, to ampicillin, penicillin and cefixime. Additionally, 42% of these strains exhibited resistance to erythromycin. *S*. *pyogenes* displayed resistance to aminoglycosides, trimethoprim/sulfamethoxazole, lomefloxacin, penicillin, vancomycin and erythromycin[76]. As mentioned by Fontes *et al*., *S*. *aureus* was the most frequently isolated bacterium and these bacterial isolates demonstrated susceptibility to nearly all antibiotics tested, albeit some showed resistance to penicillin and ampicillin + sulbactam [77]. Concerning facultative anaerobes, all tested bacteria were sensitive to gentamicin, with Gram-negative bacteria displaying a higher frequency of resistance to penicillin and clindamycin compared to Gram-positive ones [77]. Furthermore, Antonio *et al*. found that 40% of patients with *L*. *braziliensis* lesions had superinfections, with *S*. *aureus* being the most frequently isolated bacterium. The isolates exhibited resistance to antibiotics such as clindamycin and gentamicin [78]. Overall, treatment necessitates broad-spectrum antibiotics for Gram-negative bacteria and specific treatment for Gram-positive bacteria, either prior to or concurrently with antimony administration for patients with CL. Some studies have shown improved prognosis with this approach [76,79]. In summary, these findings emphasize the intricate interactions between skin microbiota and CL, underscoring the importance of ongoing research to fully grasp these relationships and their impact on disease pathogenesis.

Contrary to our observations, some studies have documented an upsurge in the abundance of the Corynebacteriaceae family in areas afflicted with skin lesions (Figs 3C and 4B). This escalation has been associated with the presence of potentially pathogenic species, notably *Corynebacterium kroppenstedii* and *Corynebacterium simulans*, particularly within the deeper layers of the skin [80–82]. Moreover, an augmentation in this family has been noted in larger lesions (>2 cm) in instances of CL induced by *L*. *donovani*, a contrast to our findings wherein no notable variations in prokaryotic and eukaryotic diversity were discerned, irrespective of the lesion’s size [27] (Figs S5 and S10 and Table 1). This discrepancy is likely attributed to the presence of *L*. *braziliensis* either in mono or coinfection in all lesions under study, a crucial factor necessitating consideration when establishing such associations. This underscores the imperative for inter-species comparisons to gain a deeper understanding of these correlations. Conversely, the reduction in the abundance of Propionibacteriaceae family members has been correlated with a decline in inflammatory processes within skin ailments of a similar nature [62,83] (Fig 4C). Further research is essential to uncover the interactions among microbiota, *Leishmania* and hosts, in order to understand how this influence the evolution of CL. This knowledge will be pivotal in exploring potential treatments for CL.

At the genus and species levels of prokaryotes, our study did not reveal significant differences in abundance or diversity between the lesion and healthy skin. This lack of distinction may be attributed to various factors, such as the infecting *Leishmania* species or environmental conditions, which could act as modifying elements (S12 Fig). However, previous research has highlighted certain genera like *Trabusiella*, *Micrococcus* and *Corynebacterium*, which demonstrate higher abundances in LCL lesions caused by *L*. *donovani*. This trend is particularly noticeable when lesions are more humid, larger in size (>2 cm) and have a greater parasite load [27] (Table 1). In the case of the *Acinetobacter* genus, where our study showed a weak association between abundance values and changes in parasite load (Fig 5), it is essential to consider that certain members of this genus, such as *Acinetobacter baumannii*, are clinically significant pathogens due to their antibiotic resistance profile. Additionally, they have been identified as prevalent components of bacterial communities on the skin associated with arthropod bites [84–86]. Hence, upcoming studies should focus on isolating this genus from CL ulcers, examining its antimicrobial resistance profile, and thoroughly assessing its role in various aspects of the disease’s pathogenesis.

Related to our findings, when considering variables such as lesion size, parasite load (except for the *Acinetobacter* genus) (S15 Fig), phototypes, geographic location of the samples and the body area sampled, we did not observe significant differences in alpha or beta diversity (S2–S11 Figs), in contrast to results reported by Kaluarachchi *et al*. [27], probably due to different *Leishmania* species presence. Additionally, existing literature indicates that microbiota changes are not necessarily linked to skin phototype, aligning with our results in both prokaryotes and eukaryotes (S3 and S8 Figs). Recent studies suggest that, rather than being influenced by skin phototype, the microbiota is altered by exposure to sunlight. It has been observed that increased sun exposure (a variable not assessed in this study) is associated with a decrease in the abundance and diversity of Proteobacteria. Moreover, these changes tend to reverse approximately a month after adequate sun protection measures are taken [87]. However, there are still several gaps that need to be addressed to better understand how the microbiota influences skin diseases and identify the factors that affect the stability or dysbiosis of the skin environment.

In the realm of eukaryotes, Ereqat *et al*. shed light on LCL lesions caused by *L*. *major* and *L*. *tropica*, being among the few studies that highlight an augmentation in the abundance of Saccharomycetales and a reduction in Exobasidiomycetes, aligning with our findings (Fig 3D) [26]. Members of the Saccharomycetales family, notably *Saccharomyces cerevisiae*, have been extensively researched for their role in the intestinal microbiome, impacting melanogenesis and the transfer of melanosomes in the skin. Furthermore, they are linked to the localized regulation of inflammatory processes in immune-mediated ailments like psoriasis [88,89]. Notably, in our eukaryotic analysis, there was an upsurge in the abundance of the species *Malassezia restricta* and a decline in other *Exobasidiomycetes sp*. (Figs 3F and 4D). *Malassezia restricta* was notably abundant in lesions caused by *Leishmania sp*. and has been reported in higher proportions in other irritative skin conditions, indicating an exacerbation of the disease [90–93]. These conditions encompass seborrheic dermatitis and psoriasis, attributable to the production of lipases in areas of the body with a high density of pilosebaceous units [94,95]. Conversely, members of the genus *Exobasidiomycetes sp*. have been identified within the occupational mycobiome [96], yet have not been specifically tied to particular pathologies. Nonetheless, further comprehensive studies are imperative to explore eukaryotes as an integral component of the microbiota, given their potential implications and modifying factors in disease progression. This is considering recent studies that demonstrate how eukaryotes can influence the immune response and inflammation in various anatomical niches.

In this context, a primary challenge in addressing chronic wounds lies in tackling infections and associated pathological inflammation [97]. Chronic wounds, even if not always infected, can be colonized by specific microbiomes that might induce infection or impede wound healing. While chronic wounds are polymicrobial in nature, they demonstrate lower diversity compared to healthy skin [97,98]. Remarkably, there exists a significant variability in wound microbiota among patients that cannot be solely attributed to individual factors or wound etiology. Thus, it is crucial to develop statistical models capable of considering this interpatient variability when analyzing the microbiota of chronic wounds, such as those in LCL [99,100].

While traditional culture-based studies have limitations in capturing the complete diversity, recent culture-independent studies in the last decade have shed light on the diverse microbiota present in wounds. Major constituents encompass *Staphylococcus spp*., *Pseudomonas spp*., *Corynebacterium spp*., *Streptococcus spp*., *Anaerococcus spp*. and *Enterococcus spp*., in addition to various low-abundance taxa [100–102]. Despite significant progress, further research on wound microbiota is imperative to grasp their potential contribution to the pathophysiology of lesions. Certain components of the wound microbiota could be correlated with the improvement or deterioration of the wound [98,102–105]. This is a visionary concept that could aid in the development of innovative biotechnological alternatives for treating CL by utilizing probiotics or metabolites produced by the microbiota.

Thus, this study underscores the importance of employing novel approaches that consider various aspects of the functional layers of the skin, such as the microbiota, physicochemistry, or immunology. Each of these factors plays a pivotal role in maintaining the skin barrier and could potentially serve as a focal point in the treatment or management of skin-associated diseases. Ultimately, while this study represents a snapshot in time and does not capture longitudinal changes, it lays a robust foundation for comparing and associating with intrinsic and extrinsic variables of individuals that could modify these patterns of variation in the skin microbiota, including potentially pathogenic microorganisms. Additionally, the next-generation sequencing approach utilized in this study holds promise as a valuable tool to investigate modifiers of the natural course of infectious diseases. Nonetheless, further studies are imperative to unravel the effects of coinfection by various *Leishmania* species on the microbiota and to determine if this also impacts the clinical progression of the disease and the corresponding immune response.

In conclusion, this study provides valuable insights into the complex interplay between Leishmaniasis and skin microbiota. It identifies spatial-temporal patterns in the distribution of *Leishmania* species and highlights the potential emergence of *L*. *naiffi*, adding nuance to our understanding of species prevalence. Moreover, our findings reveal significant alterations in the skin microbiota of CL lesions, demonstrating changes in microbial diversity and the relative abundance of specific bacterial families linked to CL. The study underlines the importance of considering a more extensive array of variables and techniques in future research, such as single-cell whole genome sequencing of *Leishmania*, metagenomics, culturomics and metabolomic analysis of CL lesions, to further unravel the intricate relationship between Leishmaniasis and the skin microbiota. This multidisciplinary approach holds the promise of advancing our understanding of CL pathogenesis and opening new avenues for potential treatment strategies. While this research represents a significant step forward, it also emphasizes the ongoing journey to explore and refine this field of study fully.

This study exhibits certain limitations that warrant consideration. The overlap of sampling areas with regions of high disease incidence and the relatively small sample size, along with potential oversampling in specific locations, such as Bonza in the central region of the country, could introduce bias and limit the generalizability of our findings. These factors might lead to an overrepresentation of certain *Leishmania* species, particularly *L*. *naiffi*. To gain a more comprehensive understanding of the spatial and temporal distribution of *Leishmania* species within the military population, future investigations should employ larger, more evenly distributed sample sizes. Regarding alterations in skin microbiota, while some of our results align with previous research, variations in the relative abundance of certain phyla and families raise questions. These differences may be attributed to specific factors, such as the *Leishmania* species involved, lesion size, or other unconsidered variables. Thus, additional studies are imperative controlling variables more stringent to unravel the complex interactions between microbiota and cutaneous leishmaniasis fully and address these limitations. Future studies should include several critical components, such as single-cell whole genome sequencing of *Leishmania*, estimating the microbiome through metagenomics and culturomics and evaluating the metabolome of CL lesions.

## Supporting information

S1 Fig Geographical sampling locations. Map illustrating the geographical locations from which samples were collected in Colombia. Includes an explanation of the sample codification system, which is based on the initials of the collection sites (B = Bonza, G = Guaviare, M = Medellín), the sample type (L = Skin Lesion, S = Healthy Skin) and a unique numerical identifier (e.g., BL-13). The map was constructed using QGIS version 2.18.7. Basemap: Elevation/World_Hillshade https://bit.ly/3vVQ1lL; Sources: Esri, Airbus DS, USGS, NGA, NASA, CGIAR, N Robinson, NCEAS, NLS, OS, NMA, Geodatastyrelsen, Rijkswaterstaat, GSA, Geoland, FEMA.
(TIF)

S2 Fig Alpha diversity indices of prokaryotes and eukaryotes by collection sites. Alpha diversity indices of prokaryotes (A) and eukaryotes (B) based on the sample type (healthy skin (Healthy) or the lesion (Lesion)) divided by collection sites (Bonza, Guaviare, Medellín). There are not significant differences between collection sites groups.
(TIF)

S3 Fig Alpha diversity indices of prokaryotes and eukaryotes by skin phototypes. Alpha diversity indices of prokaryotes (A) and eukaryotes (B) based on the sample type (healthy skin (Healthy) or the lesion (Lesion)) divided by skin phototypes (II, III, IV, V). There are not significant differences between skin phototypes groups.
(TIF)

S4 Fig Alpha diversity indices of prokaryotes and eukaryotes by injury body location. Alpha diversity indices of prokaryotes (A) and eukaryotes (B) based on the sample type (healthy skin (Healthy) or the lesion (Lesion)) divided by injury body location. There are not significant differences between injury body location groups.
(TIF)

S5 Fig Alpha diversity indices of prokaryotes and eukaryotes by ulcer size. Alpha diversity indices of prokaryotes (A) and eukaryotes (B) based on the sample type (healthy skin (Healthy) or the lesion (Lesion)) divided by ulcer size (categorized into quartiles: Q1 = 150mm, Q2 = 151-245mm, Q3 = 246-501mm, Q4 = 502-1963mm). There are not significant differences between ulcer size groups.
(TIF)

S6 Fig Alpha diversity indices of prokaryotes and eukaryotes by *Leishmania* species. Alpha diversity indices of prokaryotes (A) and eukaryotes (B) based on the sample type (healthy skin (Healthy) or the lesion (Lesion)) divided by *Leishmania* species (*L*.*b = L*. *braziliensis*, *L*.*b/Ln*. *= L*. *braziliensis/L*. *naiffi*, *L*.*b/Ln*.*/L*.*p-g = L*. *braziliensis/L*. *naiffi/L*.*panamensis-guyanenensis* complex, *L*.*p-g = L*.*panamensis-guyanenensis*). There are not significant differences between species groups.
(TIF)

S7 Fig Beta diversity indices of prokaryotes and eukaryotes by collection sites. Beta diversity of prokaryotes (A) and eukaryotes (B) based on the sample type (healthy skin (Healthy) or the lesion (Lesion)) divided by collection sites (Bonza, Guaviare, Medellín). There are not significant differences between collection sites groups.
(TIF)

S8 Fig Beta diversity indices of prokaryotes and eukaryotes by skin phototypes. Beta diversity of prokaryotes (A) and eukaryotes (B) based on the sample type (healthy skin (Healthy) or the lesion (Lesion)) divided by skin phototypes (II, III, IV, V). There are not significant differences between skin phototypes groups.
(TIF)

S9 Fig Beta diversity indices of prokaryotes and eukaryotes by injury body location. Beta diversity of prokaryotes (A) and eukaryotes (B) based on the sample type (healthy skin (Healthy) or the lesion (Lesion)) divided by injury body location. There are not significant differences between injury body location groups.
(TIF)

S10 Fig Beta diversity indices of prokaryotes and eukaryotes by ulcer size. Beta diversity of prokaryotes (A) and eukaryotes (B) based on the sample type (healthy skin (Healthy) or the lesion (Lesion)) divided by ulcer size (categorized into quartiles: Q1 = 150mm, Q2 = 151-245mm, Q3 = 246-501mm, Q4 = 502-1963mm). There are not significant differences between ulcer size groups.
(TIF)

S11 Fig Beta diversity indices of prokaryotes and eukaryotes by *Leishmania* species. Beta diversity of prokaryotes (A) and eukaryotes (B) based on the sample type (healthy skin (Healthy) or the lesion (Lesion)) divided by *Leishmania* species. There are not significant differences between species groups.
(TIF)

S12 Fig Relative abundance of prokaryotic species. Boxplots illustrating changes in the relative abundance of prokaryotic species within samples categorized by healthy skin (Healthy) and lesion skin (Lesion). There are not significant differences between prokaryotic species groups.
(TIF)

S13 Fig Differential abundance analysis of prokaryotic phyla using the DESeq2 package. Denoting uncovered an increase in abundance for the *Firmicutes* and *Proteobacteria* phyla, owing to positive rate changes.
(TIF)

S14 Fig Microbiome Composition Analysis (ANCOM). This analysis was made with bias correction allowing to observe a set of species exhibited significant differential abundances, with prominent species including *Clostridium sp*., *Eubacterium* sp. and *Aerococcus sp*., related with information provided in S2 Table.
(TIFF)

S15 Fig Correlation analysis between prokaryotic genus and parasitic load. showing the abundance values of the prokaryotic genus, the only significant genus in this analysis was *Acinetobacter* (Fig 5). Spearman’s correlation test was applied and the resulting coefficient (R) and p-value are clearly indicated.
(TIF)

S1 Table Metadata associated to samples information. Showing sample type, location, ulcer area quartile (categorized into quartiles: Q1 = 150mm, Q2 = 151-245mm, Q3 = 246-501mm, Q4 = 502-1963mm), skin phototype, *Leishmania* species, injury location, latitude and longitude of geographical sites, parasite copies/uL and parasitic load quartile (Low (L) = 2,545 parasites/mL, Medium (M) = 2,546–14,398 parasites/mL, High (H) = 14,399–219,710 parasites/mL, Very High (VH) = 219,711–2’548,227 parasites/mL).
(XLSX)

S2 Table *Leishmania* species abundance percentages. Here are only shown those greater than 5% abundance (in relation to the total reads obtained by Oxford Nanopore Sequencing).
(XLSX)

S3 Table Microbiome Composition Analysis (ANCOM) results. Including bias correction and showing the microorganisms with a Statistical W value above the 0.7 threshold. Leading to obtain the differentially abundant species that are not due to structural cero.
(CSV)
