# Supplementary figures and images for: Prokaryotic and eukaryotic skin microbiota modifications triggered by Leishmania infection in localized Cutaneous Leishmaniasis
Source: PLoS Negl Trop Dis. 2024 Mar 13;18(3):e0012029. doi: 10.1371/journal.pntd.0012029 (PMC10962849; doi:10.1371/journal.pntd.0012029)

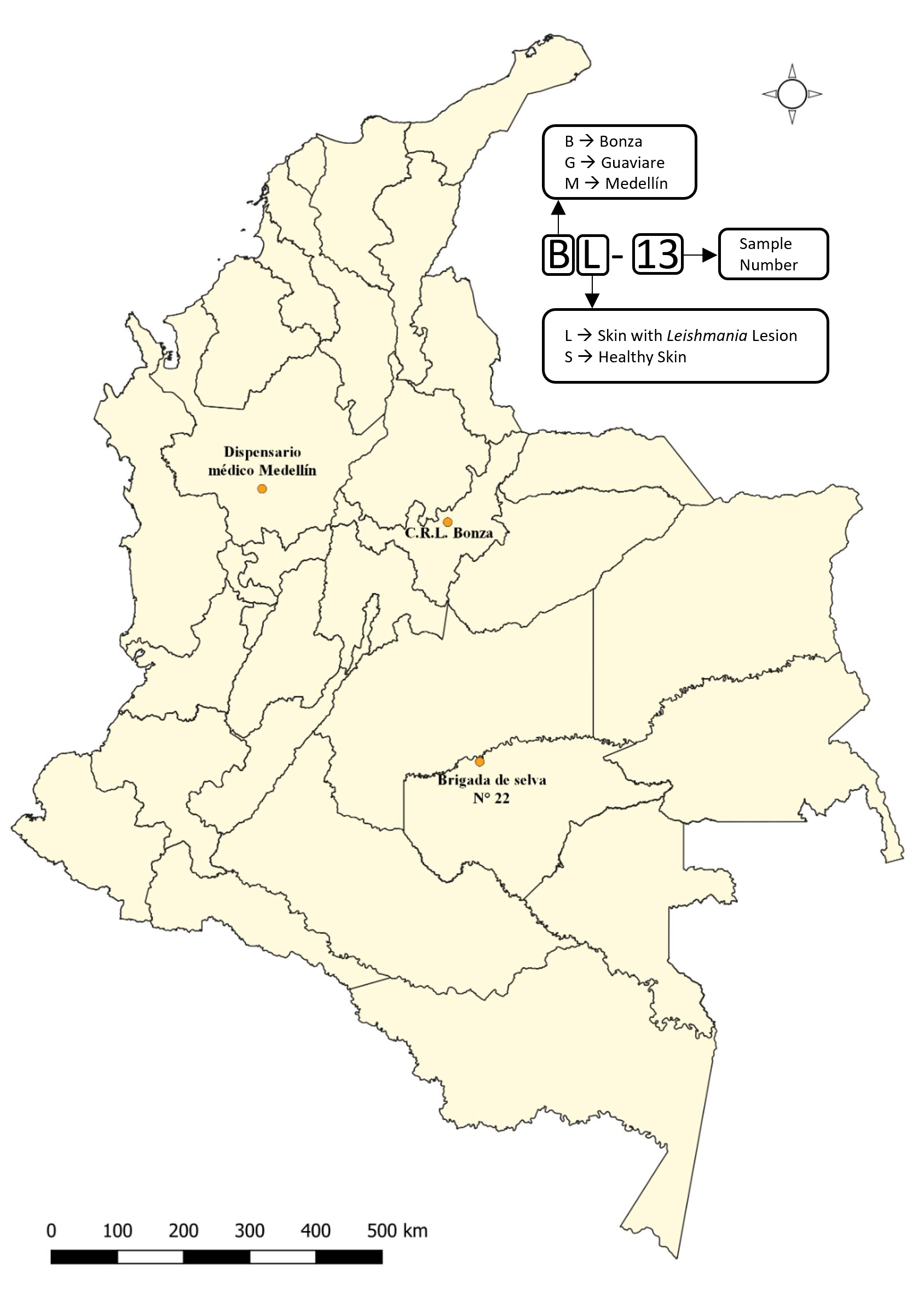

Supplement: S1 Fig — Map illustrating the geographical locations from which samples were collected in Colombia. Includes an explanation of the sample codification system, which is based on the initials of the collection sites (B = Bonza, G = Guaviare, M = Medellín), the sample type (L = Skin Lesion, S = Healthy Skin) and a unique numerical identifier (e.g., BL-13). The map was constructed using QGIS version 2.18.7. Basemap: Elevation/World_Hillshade https://bit.ly/3vVQ1lL; Sources: Esri, Airbus DS, USGS, NGA, NASA, CGIAR, N Robinson, NCEAS, NLS, OS, NMA, Geodatastyrelsen, Rijkswaterstaat, GSA, Geoland, FEMA. (TIF) [file pntd.0012029.s001.tif]

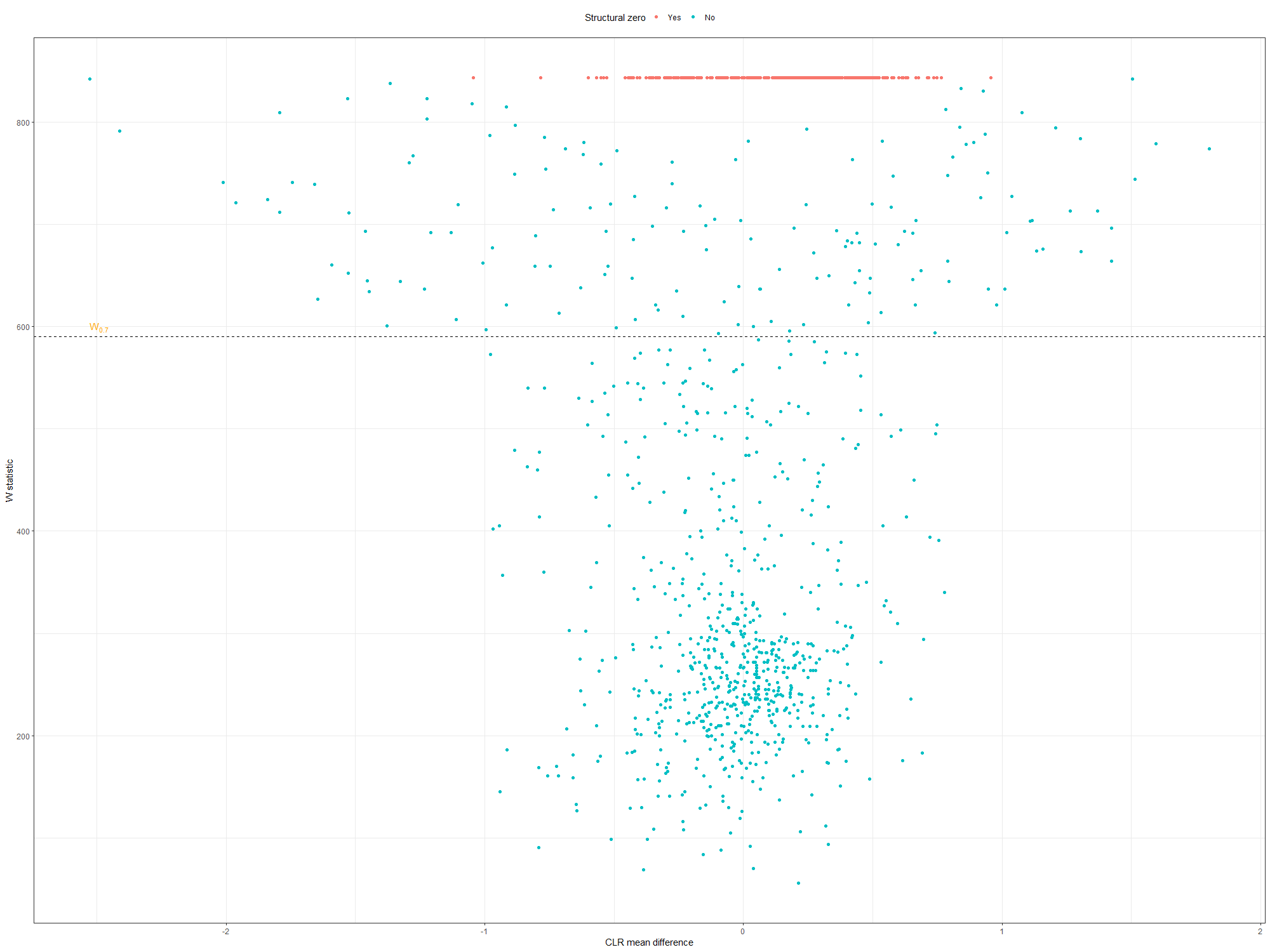

Supplement: S14 Fig — This analysis was made with bias correction allowing to observe a set of species exhibited significant differential abundances, with prominent species including Clostridium sp., Eubacterium sp. and Aerococcus sp., related with information provided in S2 Table. (TIFF) [file pntd.0012029.s014.tiff]
